# Essential Oils from Fruits with Different Colors and Leaves of *Neomitranthes obscura* (DC.) N. Silveira: An Endemic Species from Brazilian Atlantic Forest

**DOI:** 10.1155/2013/723181

**Published:** 2012-12-26

**Authors:** Raquel R. Amaral, Caio P. Fernandes, Otávio P. Caramel, Luis A. C. Tietbohl, Marcelo G. Santos, José C. T. Carvalho, Leandro Rocha

**Affiliations:** ^1^Programa de Pós-Graduação em Biotecnologia Vegetal, Centro de Ciências da Saúde, Universidade Federal do Rio de Janeiro (UFRJ), Bloco K, 2° No andar, Sala 032, Avenida Brigadeiro Trompowski s/n, 21941-590 Ilha do Fundão, RJ, Brazil; ^2^Laboratório de Tecnologia de Produtos Naturais (LTPN), Departamento de Tecnologia Farmacêutica, Faculdade de Farmácia, Universidade Federal Fluminense (UFF), Rua Mario Viana 523, Santa Rosa, 24241-000 Niterói, RJ, Brazil; ^3^Faculdade de Formação de Professores, UERJ, Rua Dr. Francisco Portela1470, Patronato, 24435-005 São Gonçalo, RJ, Brazil; ^4^Laboratório de Pesquisa em Fármacos, Colegiado de Ciências Farmacêuticas, Universidade Federal do Amapá, Campus Universitário, Marco Zero do Equador, Rodovia Juscelino Kubitschek de Oliveira, KM-02 Bairro Zerão, 68902-280 Macapá, AP, Brazil

## Abstract

*Neomitranthes obscura* (DC.) N. Silveira is an endemic plant of Brazilian Atlantic Forest and widely spread in the sandbanks of “Restinga de Jurubatiba” National Park. It is popularly known by local population as “camboim-de-cachorro” or “cambuí-preto” and recognized by its black ripe fruits. However, specimens with yellow ripe fruits were localized in the “Restinga de Jurubatiba” National Park. The aim of the present study was to evaluate chemical composition of essential oils obtained from leaves and fruits of *N. obscura* specimens with different fruit color (black and yellow) by GC and GC-MS. Essential oils from leaves of specimens with black and yellow fruits indicated a predominance of sesquiterpenes (81.1% and 84.8%, resp.). Meanwhile, essential oil from black fruits presented a predominance of monoterpenes (50.5%), while essential oil from yellow fruits had sesquiterpenes (39.9%) as major substances. Despite previous studies about this species, including essential oil extraction, to our knowledge this is the first report on *N. obscura* fruits with different colors. Our results suggest the occurrence of unless two different varieties for this species.

## 1. Introduction

“Restinga” is a type of habitat originated from Quaternary marine deposits and is represented by herbaceous and arbustive-arboreal vegetation covering typically sandy soils [1, 2]. It is characterized by large sandy coastal plains of sedimentary origin that are rippled by rows of dunes isolating lagoons, lakes, ponds, bogs, and marshes. Such a several physical conditions give rise to a diversity of habitats that are colonized by a great variety of vegetal communities [3]. “Restinga” contains many species in common with the Atlantic forest but presents diverse physiological responses to a drier habitat [4].

On this context, “Restinga de Jurubatiba” National Park is an area for permanent preservation of “restinga” habitats (Brazilian sandy coastal plain vegetation) on Rio de Janeiro State, Brazil. This area (22° to 22° 23′ S and 41° 15′ to 41° 45′ W) comprises the municipalities of Macaé, Carapebus and Quissamã [2].

The Myrtaceae family has a great diversity, mainly found across tropical and temperate areas of the globe. It comprises about 4630 species, distributed among about 144 genera [5]. In Brazil, this family is mainly constituted by wood species, being one of the dominant families in Atlantic Forest [6], and it is represented by about 23 genera and 976 species, of which 749 are endemic [7]. Despite the high number of Myrtaceae species found at “restinga” habitats and numerous voucher specimens deposited at herbaria, intricacy related to identification of many species from this family remains [8].

Chemical composition of essential oils from some Myrtaceae species from the “Restinga de Jurubatiba” National Park was evaluated. Meanwhile, essential oil from *Eugenia sulcata* Spring ex Mart. and *Myrciaria floribunda* (H.West ex Willd.) O.Berg exhibited anticholinesterase activity [9, 10].

Many species from this family are cultivated due to their edible fruits, source of scents, essences, and as ornamentals, such as Eucalyptus spp. *Eugenia uniflora* L., *Psidium guajava* L. and *Syzygium jambos* (L.) Alston [11]. In addition, numerous species from this family are used in folk medicine, such as *Psidium gajava*, which is used as antiparasitic, anti-inflammatory, antimicrobial, and treatment of intestinal diseases [12]. Ethnobotanical studies conducted with another species from this family in “restinga” areas indicate several popular uses for them, such as the treatment of diarrhea, sore throat, gout, rheumatism, influenza, urinary tract diseases, diarrhea and diabetes [13]. Species from this family also have great ecological value, since they provide important reward and attract pollinators [14, 15].

The genus *Neomitranthes* is restricted to Brazil, with sixteen identified species [7]. *Neomitranthes obscura* (DC.) N. Silveira is an endemic species of the Brazilian Atlantic Forest and widely spread in the sandbanks of “Restinga de Jurubatiba” National Park [16]. It is popularly known by local population as “camboim-de-cachorro” or “cambuí-preto” and commonly used for intestinal disorders as well as food [17, 18]. 

In the literature, ripe fruits of *Neomitranthes obscura* are described with black pericarp [8, 19]. However, specimens with yellow ripe fruits were localized during this study in the “Restinga de Jurubatiba” National Park. Both populations occur together, and there are not vegetative distinctions. This species is easily identified by the green cylindrical galls on the apical/axial bud leaf [20] and the globoses fruits crowned by the calyx tube [8]. Fruit color varieties can be found in some Myrtaceae species, but according to Moreno [21], varieties are not easily definable entities in this family and are not widely accepted.

The aim of the present study was to evaluate chemical composition of essential oils obtained from specimens of *Neomitranthes obscura* (DC.) N. Silveira with different fruit color.

## 2. Materials and Methods

### 2.1. Plant Material

Leaves and ripe fruits of *Neomitranthes obscura* (DC.) N. Silveira were collected from three individuals of each fruit color specimens in “Restinga de Jurubatiba” National Park (Rio de Janeiro, Brazil), in open *Clusia* scrub vegetation (Black fruits specimens: S 22° 13′ 4.32′′-W 41° 35′ 14.18′′; S 22° 13′ 4.67′′-W 41° 35′ 13.74′′; S 22° 13′ 3.92′′-W 41° 35′ 13.28′′/Yellow fruits specimen: S 22° 13′ 4.43′′-W 41° 35′ 14.83′′; S 22° 13′ 4.00′′-W 41° 35′ 13.96′′; S 22° 13′ 4.04′′-W 41° 35′ 14.17′′). The ripening of the fruits was characterized by the softening, sweetening, and coloring of the tissue (black or yellow pericarp in the different specimens). This species was identified by the botanist Dr. Marcelo Guerra, and voucher of the yellow fruit specimens (L. Rocha 03, 04, 06) and black fruit specimens (L. Rocha 02, 05, 07) were deposited at the herbarium of the Faculdade de Formação de Professores (Universidade do Estado do Rio de Janeiro, Brazil).

### 2.2. Extraction of the Essential Oils

Fresh leaves from the black fruit specimen (LBF) (2.720 kg), fresh leaves from the yellow fruit specimens (LYF) (2.790 kg), fresh black fruits (BF) (0.896 kg), and fresh yellow fruits (YF) (0.914 kg) were individually ground with distilled water using an automatic blender (Ética Equipamentos Científicos S.A., Brazil). Hydrodistillation method was employed using Clevenger type apparatus, and each plant material was placed in a 5 L flask [9]. The extraction was performed for 4 hours, and after this period, essential oils were collected, dried over anhydrous sodium sulphate, and stored at 4°C for further analyses.

### 2.3. Gas Chromatography/Mass Spectrometry Analysis

Essential oils were analyzed by a QP2010 (SHIMADZU) gas chromatograph equipped with a mass spectrometer using electron ionization. The gas chromatographic (GC) conditions were as follows: injector temperature, 260°C; detector temperature, 290°C; carrier gas (Helium), flow rate 1 mL/min and split injection with split ratio 1 : 40. Oven temperature was initially 60°C and then raised to 290°C at a rate of 3°C/min. One microliter of each sample, dissolved in CH_2_Cl_2_ (1 : 100 mg/*μ*L), was injected at RTX-5 column (i.d. = 0.25 mm, length 30 m, film thickness = 0.25 *μ*m). Mass spectra were recorded at 70 eV with a mass range from *m*/*z* 35 to 450 and scan rate of 1 scan/s. The retention indices (AI) were calculated by interpolation of retention times of the substances to the retention times of a mixture of aliphatic hydrocarbons (C7-C40) (Sigma) analyzed in the same conditions [25]. The identification of substances was performed by comparison of their retention indices and mass spectra with those reported in the literature [26]. The MS fragmentation pattern of compounds was also checked with NIST mass spectra libraries. Quantitative analysis of the chemical constituents was performed by flame ionization gas chromatography (GC/FID) with a QP2010 (SHIMADZU) gas chromatograph, under the same conditions of GC/MS analysis and percentages obtained by FID peak-area normalization method.

## 3. Results and Discussions

**Figure 1:**
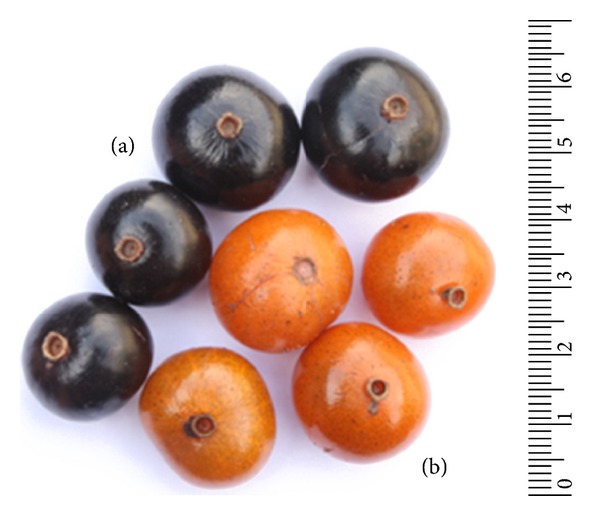
Ripe fruits of specimens from *Neomitranthes obscura* (DC.) N. Silveira in “Restinga de Jurubatiba” National Park. (a) Black fruits specimen. (b) Yellow fruits specimen.

According to Souza and Morim [8], the species *N*. *obscura* has globoses fruits crowned by the calyx tube with black pericarp when ripe. These characteristics are helpful in the identification of this species [8]. During our study, it was observed not only specimens with the characteristic black ripe fruits, but also populations with ripe yellow fruits (Figure 1). The use of essential oils in studies of intra- and interspecific genetic diversity and geographic patterns of variation in several plant species is well recognized [27]. Thus, an investigation was performed to analyze the chemical pattern of essential oils from these different colorful fruit specimens.

After extraction, the essential oils obtained from LBF and LYF yielded 0.50% (w/w) and 0.37% (w/w), respectively. Essential oils from BF and YF yielded 0.02% (w/w) and 0.07% (w/w), respectively.

The chemical analysis performed by GC-MS/GC-FID indicated a predominance of sesquiterpenes on both leaves essential oils, corresponding to 81.1% and 84.8% of relative composition of LBF and LYF, respectively. These contents were mainly constituted by sesquiterpenes hydrocarbons. Oxygenated sesquiterpenes and monoterpene hydrocarbons also appeared on leaves oils, while aliphatic compounds appeared just on LBF specimens. 

This correlation of major constituents was not observed for each fruits analyzed. BF essential oil showed a predominance of monoterpenes (50.5%) and was constituted by 10.5% of sesquiterpenes. Meanwhile, YF essential oil presented sesquiterpenes (39.9%) as major substances and was constituted by 16.1% of monoterpenes. In all, 27 substances were identified on this essential oil. Both essential oils from fruits presented aliphatic compounds, corresponding to 0.9% and 1.4% of relative composition of BF and YF, respectively. 

Germacrene B was the major substance found in LBF essential oil, corresponding to 21.8% of relative composition. The major substances found in the essential oil from LYF were Selina-3,7(11)diene (18.7%), Trans-dauca-4,(11),7-diene (13.9%), and 2,4-di-tert-butylphenol (13.4%), while germacrene B corresponded to 8.4% of relative composition of this essential oil. On another study carried out with leaves from *N*. *obscura*, De Ramos et al. [28] showed that sesquiterpenes corresponded to 87% of relative composition of this essential oil, which is in accordance with our results.

*β*-pinene (13.5%) and *α*-pinene (11.0%) were the major substances found in the essential oil from BF, however, appeared in lower amounts in the essential oil from YF (*β*-pinene, 3.2%/*α*-pinene, 1.8%). Caryophyllene oxide, which appeared as the major substance in the essential oil from YF (12.6%), corresponded to 1.7% of relative composition of essential oil from BF. The substances found in the essential oils from leaves and fruits of *N*. *obscura* are presented in Table 1.

In the tropical and subtropical zones, fruits mainly consumed by mammals are often yellow or orange, while fruits consumed by birds are often red or black [29, 30]. The occurrence of two different colors for a fruit species may increase its dispersal. On another study, Gomes et al. [4] concluded that availability peak of caloric fruits coincides with energy-demanding seasons for resident and nonbreeding birds in “Restinga de Jurubatiba” National Park, despite the fact that there was no mention of different fruit colors. In addition, regarding the literature data, we can observe that essential oil from *N*. *obscura* was investigated but without any mention of differences in fruit color [28].

## 4. Conclusions

Varieties are not easily definable entities in the Myrtaceae family; however, a study carried out for different coloring fruits of *Eugenia brasiliensis* Lam. indicated that analysis of its volatiles corroborated the concept of different varieties for this species [21]. To our knowledge, this is the first report about different fruit colors of *N*. *obscura*. It is interesting from a chemical overview that the content of predominant volatiles from the two analyzed fruits had an inverse relation between sesquiterpenes and monoterpenes. These results suggest that preferable metabolic production of monoterpenes or sesquiterpenes is followed for each specimen. Thus, the color and chemical constituents of different fruits of *N*. *obscura* suggest that this Myrtaceae species contains at least two different varieties.

## Figures and Tables

**Table 1:** Chemical constituents of essential oil from leaves and fruits of *Neomitranthes obscura *(DC). N. Silveira.

		Plant part			
Compound	AI	Leaves		Fruits	
		Black	Yellow	Black	Yellow
Hexanal	803	—	—	—	0.6
Unidentified^1^	812	0.3	1.7	—	—
Unidentified^2^	854	—	—	1.8	0.8
*α*-Pinene	936	0.9	1.7	11.0	1.8
Unidentified^3^	954	—	—	0.9	—
(3Z)-Octen-2-ol	968	—	—	0.9	—
*β*-Pinene	980	1.2	2.0	13.5	3.2
Myrcene	993	0.5	—	1.6	—
*α*-Phellandrene	1008	0.4	—	—	—
*δ*-3-Carene	1013	1.0	2.1	—	—
o-Cymene	1027	—	—	0.7	0.6
Limonene	1031	1.3	2.7	2.4	0.7
Unidentified^4^	1106	—	—	—	0.6
*α* -Camphollenal	1129	—	—	1.3	0.6
Trans-pinocarveol	1142	—	—	3.4	1.5
Trans-verbenol	1148	—	—	1.0	0.7
Pinocarvone	1166	—	—	1.0	—
p-Cymen-8-ol	1188	—	—	2.6	2.0
*α*-Terpineol	1194	—	—	4.1	1.7
Myrtenol	1200	—	—	3.0	1.5
Verbenone	1213	—	—	1.2	—
1,3,3-Trimethyl-2-oxabicyclo[2.2.2]octan-6-ol [22]	1226	—	—	1.3	—
Unidentified^5^	1278	—	—	1.2	—
Unidentified^6^	1317	—	—	2.6	—
Trans-p-menth-6-en-2,8-diol	1381	—	—	1.2	—
Sativene	1397	3.1	2.4	—	1.2
Unidentified^7^	1403	—	—	2.4	—
(E)-Caryophyllene	1424	7.0	6.2	—	—
Carvone hydrate	1428	—	—	1.2	—
*γ*-Elemene	1437	2.3	—	—	—
*α*-Guaiene	1443	1.5	—	—	—
*α*-Humulene	1458	0.6	—	—	—
*γ*-Gurjunene	1481	1.0	—	—	—
Unidentified^8^	1485	—	—	—	0.9
*β*-Selinene	1491	6.3	6.0	1.6	2.1
Unidentified^9^	1496	1.9	—	—	—
Unidentified^10^	1498	—	—	1.9	—
*α*-Selinene	1500	6.4	5.7	—	—
*α*-Bulnesene	1510	1.1	—	—	—
*β*-Bisabolene	1512	—	3.4	—	—
2,4-di-tert-butylphenol [23]	1516	1.9	13.4	—	—
7-epi-*α*-Selinene	1523	2.5	2.3	—	—
(E)-*γ*-Bisabolene	1536	—	4.4	—	—
Selina-3,7,(11)diene	1540	14.1	18.7	—	—
Unidentified^11^	1543	4.7	5.0	—	—
Trans-dauca-4 (11)7-diene	1547	11.4	13.9	—	—
Unidentified^12^	1558	—	—	—	0.8
Germacrene B	1563	21.8	8.4	—	—
Unidentified^13^	1568	0.8	—	—	—
Caryophyllene oxide	1589	—	—	1.7	12.6
Unidentified^14^	1594	—	—	—	2.5
Humulene epoxide II	1615	—	—	—	2.2
Unidentified^15^	1619	—	—	—	1.5
Unidentified^l6^	1624	0.9	—	—	—
Unidentified^17^	1633	—	—	1.9	2.6
Unidentified^18^	1638	—	—	—	1.3
Desmetoxy encecalin	1650	—	—	2.3	3.6
*β*-Eudesmol	1656	—	—	—	1.0
Unidentified^19^	1658	0.6	—	—	0.6
Unidentified^20^	1659	0.2	—	—	—
Selin-11-en-4*α*-ol	1661	1.2	—	3.5	6.0
Allohimachalol	1663	—	—	1.4	—
Unidentified^21^	1671	0.7	—	—	—
*α*-(z)-Santalol	1677	—	—	—	2.0
Cadalene	1681	—	—	—	1.2
Unidentified^22^	1687	—	—	—	0.9
Unidentified^23^	1689	—	—	—	1.1
Unidentified^24^	1693	—	—	1.3	1.2
Juniper camphor [24]	1702	—	—	—	1.1
Unidentified^25^	1723	—	—	—	1.3
Unidentified^26^	1733	—	—	—	1.5
Cyclocolorenone	1757	—	—	—	1.9
Unidentified^27^	1764	—	—	3.5	1.1
Aristolone	1768	—	—	—	2.1
Unidentified^28^	1778	—	—	—	1.7
Unidentified^29^	1791	—	—	4.1	4.5
Unidentified^30^	1796	—	—	—	0.7
Unidentified^31^	1801	—	—	—	0.9
Unidentified^32^	1804	—	—	—	1.2
Unidentified^33^	1808	—	—	—	1.2
Unidentified^34^	1814	—	—	1.98	3.1
Unidentified^35^	1826	—	—	—	2.4
Unidentified^36^	1872	0.8	—	—	—
Unidentified^37^	1875	—	—	—	1.2
Unidentified^38^	1879	—	—	2.0	—
Unidentified^39^	1906	—	—	1.2	2.0
Unidentified^40^	1910	—	—	—	1.1
Unidentified^41^	1924	0.8	—	2.4	—
Unidentified^42^	1952	—	—	4.1	—
Unidentified^43^	1955	—	—	—	5.1
Unidentified^44^	1963	—	—	2.1	1.6
Unidentified^45^	1967	—	—	2.9	1.9
Unidentified^46^	2034	—	—	—	1.0
Unidentified^47^	2347	0.7	—	—	—

Total identified		86.1	93.3	70.0	70.2

^1^MS *m/z* (Relat. int.): 40; 41; 45 (100); 69; 71; 87.

^2^MS *m/z* (Relat. int.): 41; 43 (100); 69; 83; 97; 98.

^3^MS *m/z* (Relat. int.): 55 (100); 69; 70; 97; 98; 112.

^4^MS *m/z* (Relat. int.): 41; 43; 57 (100); 70; 96; 98; 114; 207.

^5^MS *m/z* (Relat. int.): 41; 43; 69; 83 (100); 97; 125; 135; 207.

^6^MS *m/z* (Relat. int.): 41; 43 (100); 59; 71; 84; 100; 123.

^7^MS *m/z* (Relat. int.): 41; 43 (100); 59; 71; 95; 109; 123; 133; 151; 166.

^8^MS *m/z* (Relat. int.): 41; 55; 67; 79; 93; 107; 121; 135; 145; 163; 178 (100); 2004.

^9^MS *m/z* (Relat. int.): 41; 57; 71; 91; 105; 119; 133; 147; 161 (100); 175; 189; 204.

^10^MS *m/z* (Relat. int.): 41; 43; 59; 71; 97; 108; 112; 126; 155; 189; 204.

^11^MS *m/z* (Relat. int.): 41; 55; 67; 81; 91; 105; 119; 133; 147; 161; 175; 189 (100); 204.

^12^MS *m/z* (Relat. int.): 41; 69; 79 (100); 91; 106; 121; 135; 149; 159; 177; 187; 205; 220.

^13^MS *m/z* (Relat. int.): 41; 55; 69; 79; 91; 75; 119; 133; 148; 161; 189 (100); 204.

^14^MS *m/z* (Relat. int.): 41; 55; 67; 79; 91; 108; 119; 135; 147; 163; 178 (100); 192; 202; 220.

^15^MS *m/z* (Relat. int.): 41; 43 (100); 67; 81; 95; 109; 123; 135; 147; 161; 178; 189; 204; 222.

^l6^MS *m/z* (Relat. int.): 41; 43; 67; 81; 93; 105; 123 (100); 135; 147; 161; 189; 204.

^17^MS *m/z* (Relat. int.): 40; 44; 69; 81; 91; 105; 120; 131; 145; 159; 173; 187; 202 (100).

^18^MS *m/z* (Relat. int.): 41; 43; 59; 79; 91; 105; 119; 135; 145 (100); 161; 177; 185; 204; 218.

^19^MS *m/z* (Relat. int.): 41; 69; 81; 95; 100; 125 (100); 135; 147; 164; 177; 187; 205; 220.

^20^MS *m/z* (Relat. int.): 44; 55; 81; 93; 107; 133 (100); 149; 161; 173; 187; 202.

^21^MS *m/z* (Relat. int.): 41; 43; 67; 81; 93; 105; 122; 133; 148; 161; 189 (100); 104.

^22^MS *m/z* (Relat. int.): 41; 43; 69 (100); 79; 93; 109; 123; 133; 151; 160; 175; 187; 207; 220; 236.

^23^MS *m/z* (Relat. int.): 41; 55; 67; 81; 96 (100); 107; 123; 126; 149; 165; 179; 194; 205; 222; 252.

^24^MS *m/z* (Relat. int.): 41; 43 (100); 69; 71; 93; 109; 121; 153; 167; 178; 196.

^25^MS *m/z* (Relat. int.): 41; 43; 67; 79; 91; 105; 123; 138; 151 (100); 161; 179; 194; 205; 218.

^26^MS *m/z* (Relat. int.): 41; 43 (100); 67; 83; 93; 111; 123; 135; 149; 161; 175; 193; 208; 221; 236.

^27^MS *m/z* (Relat. int.): 41; 43; 60 (100); 73; 85; 99; 115; 129; 143; 157; 171; 185; 199; 228.

^28^MS *m/z* (Relat. int.): 41; 43 (100); 67; 79; 93; 108; 119; 138; 145; 161; 176; 194; 208; 218; 231.

^29^MS *m/z* (Relat. int.): 41; 59; 81; 95; 105; 119; 133; 147; 161; 179 (100); 187; 205; 220.

^30^MS *m/z* (Relat. int.): 41; 55; 67; 79; 96 (100); 107; 121; 135; 149; 162; 176; 189; 208; 218; 234.

^31^MS *m/z* (Relat. int.): 41; 43 (100); 59; 79; 91; 107; 123; 135; 147; 160; 177; 187; 205; 223; 232; 238.

^32^MS *m/z* (Relat. int.): 41; 67; 79; 93 (100); 108; 119; 133; 151; 161; 179; 187; 203; 221; 238.

^33^MS *m/z* (Relat. int.): 41; 43; 69; 81; 91; 107; 123 (100); 135; 145; 159; 177; 187; 205; 220; 236.

^34^MS *m/z* (Relat. int.): 41; 43; 69; 81; 95; 109; 123 (100); 135; 141; 162; 177; 191; 205; 220; 238.

^35^MS *m/z* (Relat. int.): 41; 43; 67; 83; 97; 105; 121 (100); 133; 145; 161; 176; 194; 196; 218; 236.

^36^MS *m/z* (Relat. int.): 40; 57; 76; 93; 104; 149 (100); 167; 207; 223.

^37^MS *m/z* (Relat. int.): 43 (100); 59; 71; 95; 99; 119; 137; 151; 159; 177; 193; 199; 219; 234; 252.

^38^MS *m/z* (Relat. int.): 41; 69; 81; 95; 105; 123; 135; 150; 164 (100); 179; 205; 220; 238.

^39^MS *m/z* (Relat. int.): 43; 69; 81; 97; 109; 121; 135; 150; 161; 179 (100); 197.

^40^MS *m/z* (Relat. int.): 41; 69; 79; 95; 107; 123; 138 (100); 149; 159; 173; 191; 201; 216; 234.

^41^MS *m/z* (Relat. int.): 41; 55; 57 (100); 71; 85; 99; 113; 127; 141; 155; 175; 189; 205; 217; 232; 261.

^42^MS *m/z* (Relat. int.): 41; 43; 59; 81; 97; 109 (100); 121; 135; 147; 161; 179; 191; 197; 219; 234.

^43^MS *m/z* (Relat. int.): 41; 43; 59; 81; 97; 109 (100); 121; 135; 147; 161; 179; 191; 197; 219; 233; 238.

^44^MS *m/z* (Relat. int.): 41; 43 (100); 60; 73; 87; 101; 115; 129; 143; 157; 174; 185; 199; 213; 256.

^45^MS *m/z* (Relat. int.): 41; 43; 69; 81; 97; 109; 121; 135; 149; 161; 179; 187; 197 (100); 220; 238.

^46^MS *m/z* (Relat. int.): 43; 67; 81; 93; 105; 119; 133; 149; 159; 177; 195 (100); 217.

^47^MS *m/z* (Relat. int.): 41; 55; 69; 85; 99; 113; 127; 165; 179 (100); 207; 261; 343.
